# Tomato Oxalyl-CoA Synthetase Degrades Oxalate and Affects Fruit Quality

**DOI:** 10.3389/fpls.2022.951386

**Published:** 2022-07-07

**Authors:** Pengfei Li, Qiyu He, Jianfeng Jin, Yu Liu, Yuxin Wen, Kai Zhao, Guangqun Mao, Wei Fan, Jianli Yang

**Affiliations:** ^1^State Key Laboratory of Plant Physiology and Biochemistry, College of Life Science, Zhejiang University, Hangzhou, China; ^2^College of Horticulture and Landscape, Yunnan Agricultural University, Kunming, China

**Keywords:** *AAE3* gene, oxalate, fruit quality, redox metabolism, fruit development

## Abstract

Acyl activating enzyme 3 (AAE3) encodes oxalyl-CoA synthetase involved in oxalate degradation. In this study, we investigated the role of AAE3 (SlAAE3) in the fruit quality of tomato (*Solanum lycopersicum*). The purified recombinant SlAAE3 protein from *Escherichia coli* exhibited a high activity toward oxalate, with a K_*m*_ of 223.8 ± 20.03 μm and V_*max*_ of 7.908 ± 0.606 μmol mg^–1^ protein min^–1^. Transient expression of SlAAE3-green fluorescent protein (GFP) fusion proteins suggests that SlAAE3 is a soluble protein without specific subcellular localization. The expression of SlAAE3 is both tissue- and development-dependent, and increased during fruit ripping. The *Slaae3* knockout mutants had improved fruit quality as evidenced by the increased sugar-acid ratio and mineral nutrient content. To find the mechanism by which SlAAE3 affects fruit quality, transcriptome, and metabolome were employed on SlAAE3 over-expressed line and wide type fruits. The transcriptomic and metabolic profiles indicated that SlAAE3 in fruits mainly functions at 20 days post-anthesis (20 DPA) and mature green (MG) stages, resulting in up-regulation of amino acid derivatives, nucleotides, and derivatives, but down-regulation of lipid compounds. However, differentially expressed genes (DEGs) were mainly enriched at redox pathways. Taken together, both *in vivo* and *in vitro* results suggest that SlAAE3-encoded protein acts as an oxalyl-CoA synthetase, which also participates in redox metabolism. These data provide a further understanding of the mechanism by which SlAAE3 participates in tomato fruit quality.

## Introduction

Oxalic acid is the simplest dicarboxylic acid and is classified as strong acid with two pKa values of 1.23 and 4.26 (Kumar et al., 2019). Oxalic acid exists extensively in nature and can be found in both living (animals, plants, bacteria, and fungi) and non-living bodies (rocks and soil). Owing to its strong acidity and chelation properties, oxalic acid typically exists in the form of soluble sodium or potassium oxalate and insoluble calcium oxalate (Dutton and Evans, 2011).

Many plant species can produce oxalic acid. Oxalate is thought to play an important role in maintaining calcium homeostasis. When calcium increases in the environment, more calcium oxalate is generated in the plant (Franceschi and Horner, 1979; Borchert, 1985; Franceschi, 2005). Moreover, oxalate was secreted from plant roots when the roots were exposed to aluminum (Yang et al., 2013) and heavy metals like cadmium (Zhu et al., 2011), nickel (Boyd et al., 2002), zinc, and lead (Fomina et al., 2005). Oxalate also protects plants from herbivorous insects. For instance, Korth proved the defensive role of calcium oxalate against chewing insects as evidenced by *M. truncatula* mutants with a decreased level of oxalic acid (Korth et al., 2006). By contrast, Hudgins found that the increased calcium oxalate crystal accumulation tends to be antagonistic to beetle attacks (Hudgins et al., 2003). Additionally, oxalate is able to balance excessive cations (Osmond, 1963). However, excessive oxalate will lead to multiple physiological disorders such as disturbance of mitochondrial metabolism, free radical formation, and disruption of membrane integrity (Scheid et al., 1996). In addition, oxalate is used by some pathogens during their invasion of hosts. During the invasion process, oxalate is thought to stimulate stoma cells, chelate cations, and act as a trigger of programmed cell death (Guimarães and Stotz, 2004; Dong et al., 2008; Kim et al., 2008). Due to the deleterious activities of oxalate, the content of oxalate in living bodies should be strictly regulated to maintain normal metabolic processes.

In plants, three pathways have been implicated in oxalate biosynthesis, namely, glycolate/glyoxylate pathway (Franceschi and Nakata, 2005), oxaloacetate pathway (Chang and Beevers, 1968; Dutton and Evans, 2011), and ascorbic acid pathway (Kostman et al., 2001; Franceschi and Nakata, 2005). When in excess, oxalate is also degraded by three different pathways, namely, oxidation (Svedruzić et al., 2005), decarboxylation (Datta and Meeuse, 1955), and acetylation (Foster et al., 2012), respectively. The acetylation pathway is the most well-documented pathway involving four enzymes. Oxalate is first transformed into oxalyl-CoA by oxalyl-CoA synthetase and then degraded into formyl-CoA and CO_2_ by oxalyl-CoA decarboxylase. Next, formyl-CoA is broken into formate cataylzed by formyl-CoA hydrolase. Finally, formate is degraded into CO_2_ and H_2_O by formate dehydrogenase (Foster et al., 2012, 2016; Foster and Nakata, 2014; Lou et al., 2016).

In animals, unlike plants, oxalate is generally considered to be pathological. Nearly 20% of the oxalate in animals, as well as humans, comes from the diet (Williams and Wandzilak, 1989). The soluble form of oxalate, which often exists as sodium oxalate, can be absorbed directly, contributing to renal stone disease (Holmes et al., 1995, 2001). Too much oxalate obtained from food intake can also cause heart disease, cardiac conduction disorder, and even death (Camici et al., 1982; Lindsjö et al., 1989; Rodby et al., 1991). The insoluble form of oxalate, which often exists as calcium oxalate, is acting as an antinutrient, blocking the uptake of mineral nutrients (Weaver et al., 1987; Heaney and Weaver, 1989). By expressing an oxalate decarboxylase, Kumar et al. (2016) found a reduction in oxalate content, as well as an increment of some essential micronutrients. In spinach, the decrease of oxalate correlates with the increase of ascorbates and vitamins (Joshi et al., 2021). Thus, it is promising to cultivate high-quality food with reduced oxalate content.

Tomato (*Solanum lycopersicum* L.) is rich in many nutrients such as lycopene, β-carotene, glucose, fructose, and a set of volatile chemicals. As an important fruit and vegetable, the tomato has been hugely consumed annually worldwide. It also serves as a model plant for research on fruit development and quality (Klee and Giovannoni, 2011). Besides, in the tomato plant, oxalate degradation can only be carried out through the acetylation pathway (Membré et al., 1997, 2000). Therefore, oxalyl-CoA synthetase encoded by the proposed *SlAAE3* gene, a member of AAE genes superfamily (Jin et al., 2021), must play a key role in tomato fruit quality by regulating oxalate content.

This study aims to investigate whether the tomato AAE3 has the catalytic activity toward oxalate, and if so to what extent it affects tomato fruit quality. Our study proved that *SlAAE3* encodes a cytoplasmic oxalyl-CoA synthetase involved in oxalate degradation. Comparative transcriptome and metabolome analyses revealed that *SlAAE3* exerts its role mainly at 20 days post-anthesis (DPA) and mature green (MG) stages through redox metabolism, ultimately affecting the fruit quality.

## Materials and Methods

### Plant Materials and Growth Conditions

For germination, tomato (*S. lycopersicum*) seeds were surface sterilized with 10% sodium hypochlorite for 20 min, then washed with distilled water 5 times. The seeds were planted on Petri dishes containing one-fifth strength Hoagland (pH 5.5) nutrient solution and 0.8% agar (Zhu et al., 2011). Petri dishes were placed in a growth chamber maintained at 25°C with a 16/8-h day/night cycle for 5 days. After germination, tomato seedlings were transferred to the soil. All the seedlings were placed in a greenhouse with a 12 h/25°C day (light intensity of 30000 lux) and a 12 h/23°C night regime, and relative humidity of 60%. One-fifth strength Hoagland nutrient solution was added every 2 days. According to Shinozaki, tomato fruits were classified into different development stages (Shinozaki et al., 2018). Fruits at 20 days post-anthesis (DPA), mature green (MG), pink (Pk), and red ripe (RR) stages were collected for metabolomics and transcriptomics experiments.

### Construction of Tomato Transgenic Lines

The *Slaae3* knockout mutants were generated by Biogle Co., Ltd., Hangzhou, China^1^ in the background of cv. Micro-Tom, and transgenic seedlings were confirmed by sequencing. For overexpression lines, the full-length coding sequences (CDS) of *SlAAE3-1* gene with stop codon were cloned by polymerase chain reaction (PCR) using a pair of primers (Supplementary Table 1) and ligated into binary vector 35S:GFP (modified from pCAMBIA2300). The resulting 35S:SlAAE3-1 plasmids were introduced into *Agrobacterium tumefaciens* strain GV3101, which was further introduced into Ailsa Craig tomato following the method of Sun et al. (2006). The transgenic seedlings were first screened with the kanamycin and then confirmed by PCR using the same primers mentioned above.

### Subcellular Localization

The full-length coding sequences (CDS) of *SlAAE3-1* and *SlAAE3-2* genes without stop codon were cloned by PCR using a pair of primers (Supplementary Table 1) and ligated into binary vector 35S:GFP (modified from pCAMBIA2300). The resulting 35S:SlAAE3-1:GFP and 35S:SlAAE3-2:GFP plasmids were introduced into *A. tumefaciens* strain GV3101. These positive *A. tumefaciens* strains with the resulting plasmids were transiently expressed in *Nicotiana benthamiana* by leaf infiltration. The localization of the recombinant protein was investigated using a laser scanning confocal microscope (lsm710nlo, Zeiss, Germany).

### Recombinant Protein Purification and Enzyme Activity Assay

The full-length CDS of *SlAAE3-1* and *SlAAE3-2* were obtained by PCR amplification using a pair of primers (Supplementary Table 1) and cloned into pET-28a (+) vector. The vectors were introduced into the *Escherichia coli* strain BL21 (DE3) for protein expression. Positive strains containing the resulting plasmids were induced by 0.5 mM (SlAAE3-1) and 1 mM (SlAAE3-2) IPTG at 16°C for 8 h, respectively. The cells were collected and resuspended in PBS buffer (137 mM NaCl, 2.7 mM KCl, 10 mM Na_2_HPO_4_•12H_2_O, 1.76 mM KH_2_PO_4_, pH 8.0). The recombinant proteins were collected using Ni NTA Beads 6FF under the manufacturer’s instructions and concentrated by ultrafiltration (50 kD Mr cutoff, Millipore) and then equilibrated by 0.1 M Tris-HCl (pH 8.0). The purity of the recombinant proteins was determined by the SDS-PAGE followed by Coomassie Brilliant Blue staining.

For enzyme activity assay, the assay buffer contained 3 μg of the purified recombinant protein, 0.1 M Tris-HCl (pH 8.0), 5 mM ATP, 10 mM MgCl_2_, 0.5 mM CoA, 0.4 mM NADH, 1 mM phosphoenolpyruvate, and 10 units each of pyruvate kinase, myokinase, and lactate dehydrogenase and substrate acid, in a final volume of 1 mL. In the substrate assay, 400 μM of each candidate substrate was added, and up to 800 μM oxalate was used for the SlAAE3-1 enzyme kinetic assay. The reaction rate was measured by the NADH concentration at 340 nm spectrophotometrically immediately after mixed. The consumption of NADH results in color change from purple to faint pink (Ishiyama et al., 1997).

### Nucleic Acid Isolation and Analysis

Genomic DNA was extracted from young leaves of wide type (WT) and two *SlAAE3-1* mutant lines in the background of cv. Micro-Tom using TaKaRa MiniBEST Universal Genomic DNA Extraction Kit (Cat # 9765). The *SlAAE3-1* gene in the three lines was determined *via* PCR using primers 3-1-F and 3-1-R. Actin (Solyc02g063070.2.1) was used as an internal control (Supplementary Table 1).

Total RNA was extracted using TaKaRa MiniBEST Universal RNA Extraction Kit (Cat # 9767). Around 0.3 μg total RNA was subjected to the synthesis of first-strand cDNAs by using Rever Tra Ace^®^ qPCR RT Master Mix [TOYOBO (SHANGHAI) BIOTECH Co., Ltd., Shanghai, China]. One-fifth of the cDNA products were used for qRT-PCR analysis using SYBR^®^ Green Realtime PCR Master Mix [TOYOBO (SHANGHAI) BIOTECH Co., Ltd., China]. AAE3-1-F and AAE3-1-R were the primers for qRT-PCR analysis. Actin (Solyc02g063070.2.1) was used as an internal control (Supplementary Table 1).

### Oxalate Treatment of Leaves

The treatment assay was performed according to Kumar et al. (2016). The third or fourth compound leaves counted from the cotyledon were detached from both control and mutant plants grown for 45 days. Half of the detached petioles were immediately dipped in 20 mM oxalate acid solution (pH 4.0), with the leaves above the surface. Another half of the leaves were treated as above but with distilled water (pH 4.0) as control. All the leaves were placed in a growth chamber maintained at 25°C with a 16/8-h day/night cycle for 24, 30, and 36 h.

### Determination of Organic Acids, Sugars, and Mineral Elements

Fruits at the RR stage were harvested, frozen in liquid nitrogen, ground into powder, and then stored at −80°C for further analysis. Determination of organic acids was carried out according to Lou et al. (2016). For sugar determination, about 0.3 g of each sample was added to 1 mL of deionized water and boiled for 25 min. The sample volume was then adjusted to 10 mL by deionized water, and analyzed by ion chromatography (ICS 3000; Dionex) equipped with a CarboPacTMPA1 analytical column. The mobile phase was 200 mM NaOH at a flow rate of 1.0 mL/min. For mineral element determination, the samples were weighed and digested with HNO_3_/HClO_4_ (4:1, v/v). The digested solution was analyzed by inductively coupled plasma-mass spectrometry (ICP-MS; ICAPRQICPMS, Thermo Fisher). Each sample had three biological replicates for the determination of organic acids, sugars, and mineral elements.

### Transcriptome Analysis

Transcriptome analysis was performed using Illumina HiSeq2500 from Gene *Denovo* Biotechnology Co. (Guangzhou, China). Tomato fruits were harvested from wild-type and OE-3 plants at all four development stages mentioned above. Each sample contained five fruits at the same development stage, collected from different plants and pooled together, and ground into a fine powder as a single biological repeat. Three repeats were carried out for both wild-type and OE-3 plants. For RNA-Seq analysis, raw data were filtered by fastp v0.18.0 to get high-quality clean reads as follows: (i) removing reads containing adapters, (ii) removing reads containing more than 10% of unknown nucleotides (N), (iii) removing low quality reads containing more than 50% of low quality (Q-value ≤ 20) bases. Then, an index of the reference genome was built by HISAT2.2.4 and was used to map the paired-end clean reads. The mapped reads of all samples were assembled by using StringTie v1.3.1. Fragment per kilobase of transcript per million mapped reads (FPKM) value was calculated for each transcription region to quantify its expression abundance and variations. Differentially expressed genes (DEGs) were regarded if false discovery rate (FDR) < 0.05 and absolute fold change ≥ 2, performed by DESeq2 software. The gene ontology (GO) categories of DEGs were analyzed by the Gene Ontology database.^2^ Following the annotation results and official classification, DEGs were divided into different groups according to their involvement in biological pathways and functions.

### Metabolome Analysis

Metabolome analysis was performed by Wuhan Maiwei Biotechnology Co., Ltd., Wuhan, China^3^ using the same samples used for transcriptome analysis. 100 mg powder was dissolved and extracted with 1.2 mL 70% methanol in a refrigerator at 4°C overnight. The extracts were filtrated (SCAA-104, 0.22 μm pore size; ANPEL, Shanghai, China^4^) before ultra-performance liquid chromatography/tandem mass spectrometry (UPLC-MS/MS) analysis (UPLC, SHIMADZU Nexera X2^5^; MS, Applied Biosystems 4500 Q TRAP^6^). The samples were analyzed under the following UPLC conditions: column, Agilent SB-C18 (1.8 μm, 2.1 mm*100 mm); solvent system, water (0.1% formic acid): acetonitrile (0.1% formic acid); gradient program, 95:5 V/V at 0.0 min, 5:95 V/V at 9 min, 5:95 V/V at 10 min, 95:5 V/V at 11.1 min, 95:5 V/V at 14 min; flow rate, 0.35 mL/min; temperature, 40°C, injection volume: 4 μL. The effluent was alternatively connected to an ESI-triple quadrupole-linear ion trap (QTRAP)-MS.

Linear ion trap (LIT) and triple quadrupole (QQQ) scans were acquired on a Q TRAP, AB45000 Q TRAP UPLC/MS/MS System, equipped with an ESI Turbo Ion-Spray interface, which was controlled by Analyst 1.6.3 software (AB Sciex) and operated in positive and negative ion mode. The operation parameters of ESI were as follows: ion source, turbo spray; source temperature 550°C; ion spray voltage (IS) 5500 V (positive mode)/−4500 V (negative mode); ion source gas I (GSI), gas II (GSII), curtain gas (CUR) were set at 50, 60, and 25.0 psi, respectively; the collision-activated dissociation (CAD) was high. Polypropylene glycol solutions of 10 and 100 μmol/L in the QQQ and LIT modes were used to perform the instrument tuning and mass calibration, respectively. QQQ scans were acquired from m1ultiple reaction monitoring (MRM) experiments with collision gas (nitrogen) set to medium. R3.5.0^7^ was applied for data processing. Significantly regulated metabolites between different groups were determined by variable importance in project (VIP) ≥ 1 and absolute Log2 FC (fold change) ≥ 1. The identified metabolites were annotated using the KEGG Compound database^8^ and then mapped to the KEGG pathways.^9^ Pathways involved in significant metabolites were fed into metabolite sets enrichment analysis (MSEA), and significance was determined by the hypergeometric test’s *p*-values.

## Results

### Domain and Sequence Analysis of the SlAAE3 Protein

**FIGURE 1:**
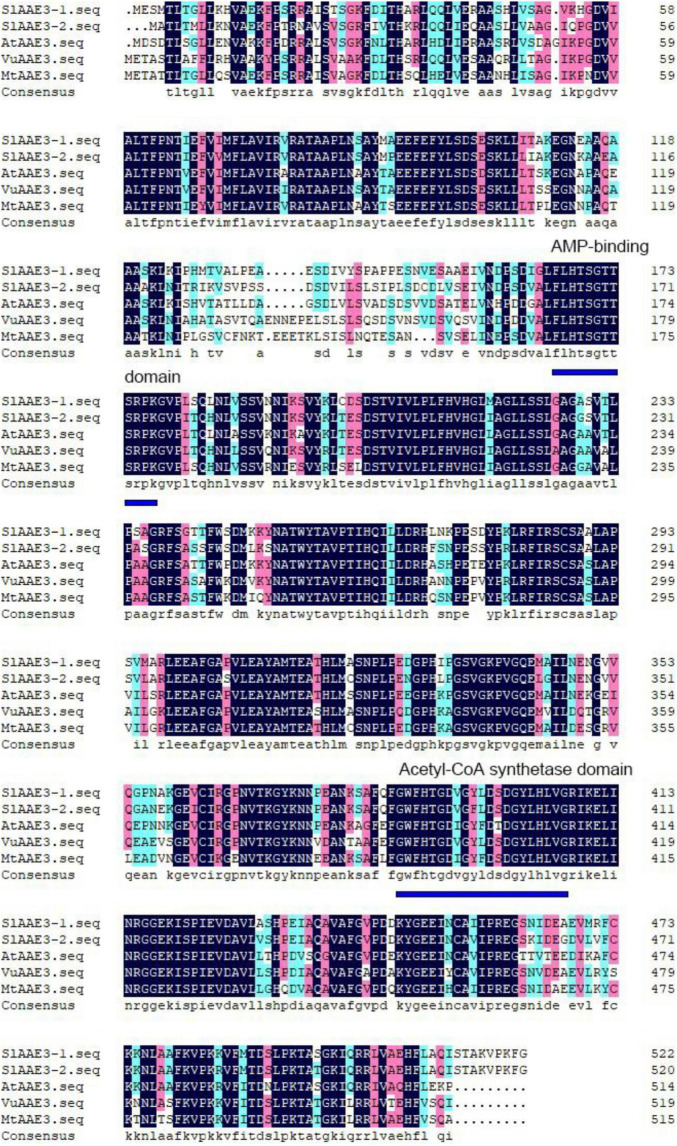
Amino acid sequence alignment of AAE3 proteins from *Solanum lycopersicum* (SlAAE3-1; Solyc03g025720.2 and SlAAE3-2; Solyc06g035960.2), *Arabidopsis thaliana* (AtAAE3; At3g48990), *Vigna umbellata* (VuAAE3; KX354978), and *Medicago truncatula* (MtAAE3; XP_003599555.1). The conserved AMP binding domain and acetyl-CoA synthetase domain are indicated by blue lines.

After BLAST of VuAAE3 protein sequence (Lou et al., 2016), two *SlAAE3* genes were found in tomato genome database,^10^ named as *SlAAE3-1* (Solyc03g025720.2.1) and *SlAAE3-2* (Solyc06g035960.2.1). *SlAAE3-1* and *SlAAE3-2* are predicted to encode a 523- and 521-amino acid protein, respectively, sharing a similarity of 82%. Both proteins contain an AMP binding domain in the N-terminal, and an acetyl-CoA synthetase domain in the C-terminal (Figure 1). Phylogenetic relationship analysis showed that the AAE3 proteins are clearly separated into two clades, the monocots and the dicots, and that SlAAE3-1 and SlAAE3-2 proteins are the most closely related to CaAAE3 from *Capsicum annuum L.* (Supplementary Figure 1). In addition, the high similarity between AAE3 proteins of *S. lycopersicum*, *Arabidopsis thaliana*, *Vigna umbellata*, and *Medicago truncatula* indicates SlAAE3-1 and SlAAE3-2 may function as an oxalyl-CoA synthetase involved in oxalate degradation process (Figure 1).

### SlAAE3-1 and SlAAE3-2 Are Localized to Both Cytoplasm and Nucleus

**FIGURE 2:**
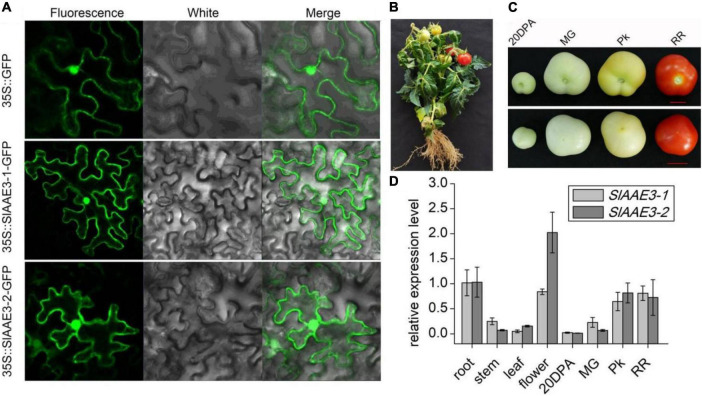
Subcellular localization and histological expression pattern of SlAAE3-1 and SlAAE3-2. **(A)** Confocal image of *Nicotiana benthamiana* cells expressing AAE3-1-eGFP or SlAAE3-2-eGFP. Bar: 50 μM. eGFP driven by 35S promoter was used as control. **(B)** Plant stature at reproductive stage. **(C)** Fruits at different development stages. 20 DPA, 20 days post-anthesis; MG, mature green; Pk, pink stage; RR, red ripe. Bar = 1 cm. Upper panel: bottom-up view of fruits. Lower panel: top view of fruits. **(D)** Relative expression of *SlAAE3-1* and *SlAAE3-2* in different organs of tomato plants. For different developmental stages of fruits, the materials were collected at corresponding stages shown in panel **(C)**. Plant materials were immediately frozen in liquid nitrogen for RNA extraction. Actin (Solyc02g063070.2.1) was used as an internal control. Data are means ± SD (*n* = 3).

Both MtAAE3 and AtAAE3 were reported to be localized in cytoplasm (Foster et al., 2012, 2016), while VuAAE3 was found to be localized in both cytoplasm and nucleus (Lou et al., 2016). In order to investigate the subcellular localization of SlAAE3-1 and SlAAE3-2, we constructed three different *A. tumefaciens* strains overexpressing SlAAE3-1-GFP fusion protein, SlAAE3-2-GFP fusion protein, or free GFP protein, under the control of strong, constitutive *Cauliflower mosaic virus* 35S promoter, respectively. The three strains were used for transient expression through infection of *N. benthamiana* leaves. Fluorescence images of *N. benthamiana* leaves expressing SlAAE3-1-GFP and SlAAE3-2-GFP fusion protein showed a strong GFP signal in the cytoplasm and nucleus, as the free GFP protein (Figure 2A). Thus, our result indicates that tomato SlAAE3-1 and SlAAE3-2 are soluble proteins, which coincides with the first step of the oxalate acetylation degradation pathway (Giovanelli and Tobin, 1961, 1964).

### SlAAE3-1 and SlAAE3-2 Are Extensively Expressed in Tomato Plant

To determine the expression pattern of *SlAAE3-1* and *SlAAE3-2* in different tissues, total RNA was extracted from the root, stem, leaf, flower (Figure 2B), and fruit (Figure 2C). The fruit was collected at four different developmental stages as described by Shinozaki: 20 DPA, MG, Pk, and RR (Shinozaki et al., 2018). *SlAAE3-1* and *SlAAE3-2* shared a similar expression pattern among the detected tissues, except flower (Figure 2D). The root tissues had higher expression of *SlAAE3-1* and *SlAAE3-2* compared with the stem and leaf. Interestingly, in the fruit, the expression of *SlAAE3-1* and *SlAAE3-2* increased with fruit growth and development. These results indicate that *SlAAE3-1* and *SlAAE3-2* are related to fruit development. What’s more, *SlAAE3-2* showed a two-fold higher expression level in the flower compared with *SlAAE3-1*, suggesting its additional functions.

### SlAAE3-1 and SlAAE3-2 Encode Oxalyl-CoA Synthetases *in vitro*

**FIGURE 3:**
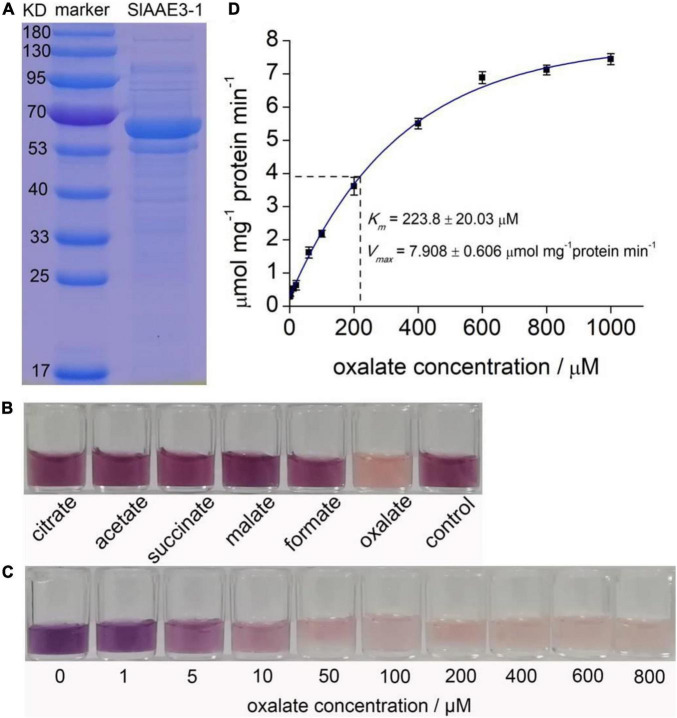
Biochemical analysis of SlAAE3-1. **(A)** SDS-PAGE gel of purified SlAAE3-1 protein (right) and molecular weight markers (left). **(B)** Reaction activity of SlAAE3-1 against different organic acids. NADH residue level indicated by nitroblue tetrazolium and 1-methoxy-5-methylphenazinium methosulfate. **(C)** Visual inspection of NADH residue level indicated by nitroblue tetrazolium and 1-Methooxy-5-methylphenazinium methosulfate. The reaction assay consumed more NADH as oxalate increased. **(D)** Kinetics assay of SlAAE3-1.

SlAAE3-1 and SlAAE3-2 are supposed to function as oxalyl-CoA synthetases as indicated by the bioinformatics analysis. To investigate their natural substrates, we purified histidine (His)-tagged fusion protein of SlAAE3-1 and SlAAE3-2 from *E. coli*. The protein was > 90% pure as showed by Coomassie Brilliant Blue staining on sodium dodecyl sulfate-polyacrylamide electrophoresis (SDS-PAGE) gel (Figure 3A). Six organic acids were used as potential substrates of SlAAE3-1 and SlAAE3-2 following Lou et al. (2016). Most of the NADH in the oxalate assay was consumed by SlAAE3-1, while negligible activities were found against the other five organic acids as indicated by Tetranitrotetrazolium blue chloride assay (Figure 3B), suggesting SlAAE3-1 should be an oxalyl-CoA synthetase specifically involved in oxalate degradation.

Then we performed enzyme kinetics analysis of SlAAE3-1 using a range of oxalate concentrations. The color shown in Figure 3C indicated that at saturating concentrations of CoA (0.5 mM) and ATP (5 mM), the consumption of NADH increased as oxalate degradation. SlAAE3-1 displayed Michaelis-Menten kinetics for oxalate concentration up to 600 μM (Figure 3D). Using this, a *V*_*max*_ of 7.908 ± 0.606 μmol min^–1^ mg^–1^ protein and a *K*_*m*_ of 223.8 ± 20.03 μM were calculated. The *V*_*max*_ of SlAAE3-1 toward oxalate is similar to VuAAE3 (Lou et al., 2016), but lower than AtAAE3 and MtAAE3 (Foster et al., 2012, 2016). However, the *K*_*m*_ for SlAAE3-1 is much higher than MtAAE3, VuAAE3, and AtAAE3 (Foster et al., 2012, 2016). Compared with SlAAE3-1, SlAAE3-2 displays a much lower activity toward oxalate degradation, though oxalate also proved to be the target substrate (Supplementary Figure 2). However, its low activity made it difficult to detect the enzyme kinetics of SlAAE3-2 by measuring the consumption of NADH.

### SlAAE3-1 Functions as an Oxalyl-CoA Synthetase *in vivo*

**FIGURE 4:**
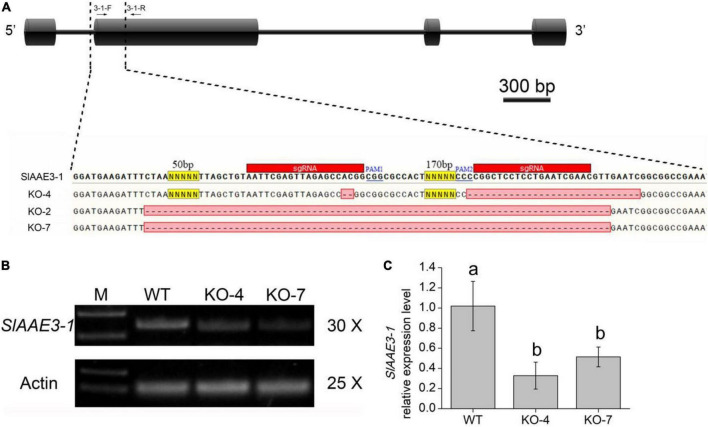
MicroTom T1 mutants were obtained *via* CRISPR/Cas9-mediated gene editing. **(A)** Gene structure and mutation sites of *SlAAE3-1*. Black boxes and horizontal lines between the boxes indicate exons and introns, respectively. Black arrows indicate PCR primers used to evaluate mutation type and efficiency. Two sgRNA were used to knock out SlAAE3-1 and the PCR products from a subset of plants were cloned and sequenced to validate the deletion in plant KO-2, KO-4, and KO-7. **(B)** PCR gel showing the type of DNA lesions generated in the genome, and **(C)** qRT-PCR analysis revealing the expression level of SlAAE3-1 of KO-4 and KO-7 plants. Error bars indicate mean values ± SD (*n* = 3). Different letters on vertical bars indicate significant difference at *p* < 0.05 using one-way ANOVA.

**FIGURE 5:**
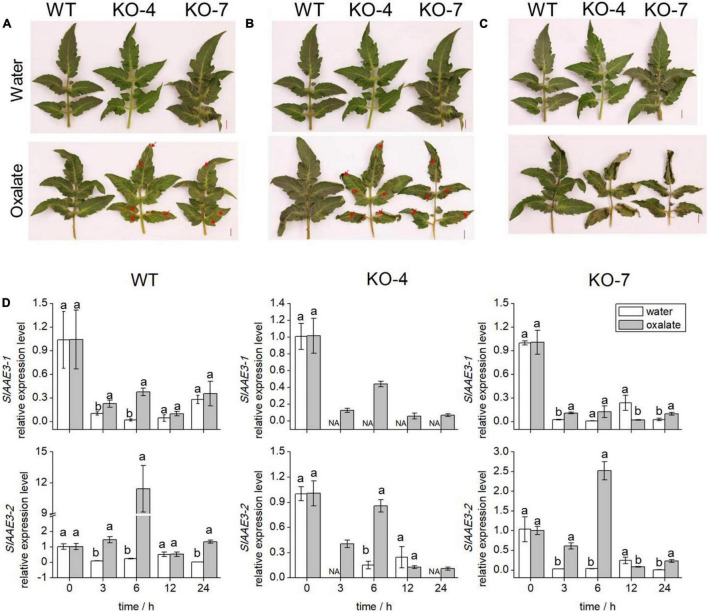
Phenotypes and transcription analyses of WT and KO mutants. The comparison of oxalic acid (OA) sensitivity between WT and KO mutants with disabled SlAAE3-1. Excised leaves were dipped into the OA solution immediately, and negative control was kept in water. The detached leaves were photographed after **(A)** 24 h, **(B)** 30 h, and **(C)** 36 h. Brown lesion and shrink area were indicated by red arrows. **(D)** qRT-PCR analyses were carried out to reveal the expression level of SlAAE3-1 and SlAAE3-2 in the detached leaves. Error bars indicate mean values ± SD (*n* = 3). Different letters on vertical bars indicate significant difference at *p* < 0.05 using one-way ANOVA.

Since SlAAE3-1 protein displayed a higher affinity toward oxalate, we developed *SlAAE3-1* knockout mutants in the background of Micro-Tom and two mutant lines were obtained, named KO-4 and KO-7. KO-4 has two mutation sites, with 2 bases deletion close to PAM1 and 27 bases deletion close to PAM2. KO-7 has a deletion of 287 bases between PAM1 and PAM2 (Figure 4A). To confirm the generations of knock-out mutants, we designed primers 3-1-F and 3-1-R located at the gaps. The PCR results showed that *SlAAE3-1* was destroyed at the genome level (Figure 4B). At the transcriptional level, the expression of *SlAAE3-1* decreased both in KO-4 and KO-7 compared with wild-type (WT) control (Figure 4C). The pinnately compound leaves of WT and the two mutant lines at the same development stages were detached and fed with oxalate following Kumar et al. (2016). After 24 h treatment with oxalate, the necrotic plaque emerged on mutant leaves (Figure 5A). As the treatment continued, the mutant lines were severely dehydrated and had increasing necrotic areas (Figures 5B,C). *SlAAE3-1* and *SlAAE3-2* could be induced by oxalate both in WT and mutant lines, however, their expression levels were lower in mutant lines compared with WT (Figure 5D).

### Knockout of *SlAAE3-1* Improved Tomato Fruit Quality

**FIGURE 6:**
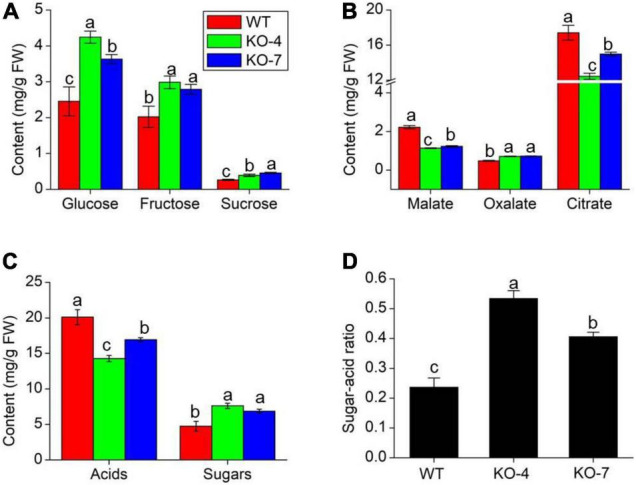
Fruit quality analysis of MT species between WT and mutants at RR stage. Main sugars **(A)**, acids **(B)**, total sugars and acids **(C)** content, and sugar-acid ratio **(D)** in WT and mutants. Error bars indicate mean values ± SD (*n* = 3). Different letters on vertical bars indicate significant difference at *p* < 0.05 using one-way ANOVA.

Oxalate has been proven to influence the levels of some micronutrients in soya bean and grass pea seeds (Kumar et al., 2016). Micronutrients are important to human health and are regarded as a key indicator of fruit quality, as well as sugar and organic acid levels (Borsani et al., 2009). We analyzed the contents of three main soluble sugars (glucose, fructose, and sucrose) and organic acids (malate, oxalate, and citrate) in tomato fruit at the RR stage. All of the sugars increased in mutant lines (Figure 6A), while the main acids decreased in mutant lines compared with WT, except oxalate (Figure 6B). It was evident that knockout of *SlAAE3-1* blocked the degradation of oxalate, but has no effects on fruit phenotype (Supplementary Figure 3). These changes resulted in higher total sugar content and lower total acid content in mutant lines compared with WT (Figure 6C). Finally, function impairment of *SlAAE3-1* resulted in about 2-fold higher sugar-acid ratio (Figure 6D). We also measured the mineral nutrients, and their concentration did not show a clear trend. However, most of the mineral nutrients increased at the RR stage, including Mg, Zn, Fe, Ca, P, and Mu (Supplementary Figure 4). These data suggest the absence of *SlAAE3-1* promotes tomato fruit quality.

### Metabolomics Profiles of Tomato Fruits at Different Maturation Stages

**FIGURE 7:**
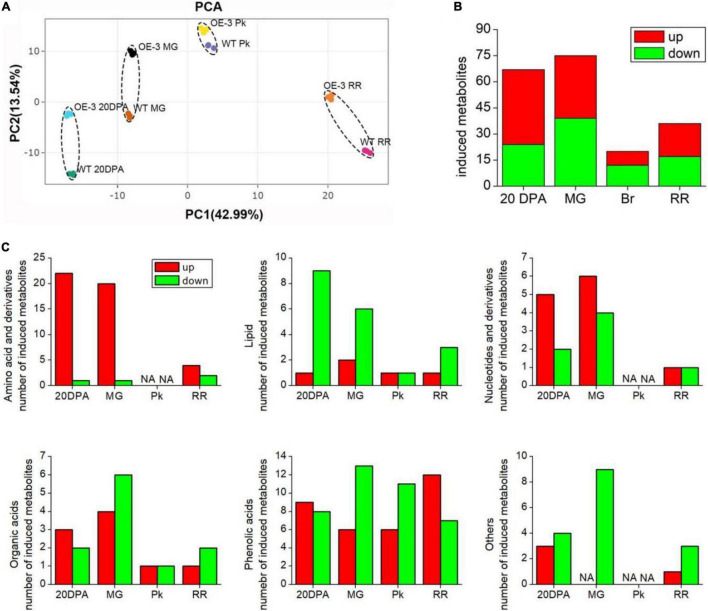
Identification of significantly changed primary metabolites in WT and OE-3 fruits at different development stages. **(A)** Principal component analysis (PCA) of primary metabolites. PCA score plots (PC1 vs. PC2) for the first two principal components after SlAAE3-1 overexpression. Each sample was marked using individual color. Statistics of induced total **(B)** and classified **(C)** metabolites at different development stages.

Considering Micro-Tom is an ornamental plant, and has low economic value, we next overexpressed *SlAAE3-1* into tomato cv. AC to analyze its function. Two independent lines were obtained, with the relative expression level of *SlAAE3-1* increased by 2.5 (OE-3) and 1.5-folds (OE-5), respectively (Supplementary Figure 5A). According to Shinozaki, we harvested the fruits of OE lines and WT at 20DPA, MG, Pk, and RR stages and carried out metabonomics analysis (Shinozaki et al., 2018). However, only the metabonomics profiles of OE-3 have a clear difference from WT, so further analysis was carried out between OE-3 and WT. No significant differences were found in shape and color between the fruits of OE-3 and WT at the same development stages (Supplementary Figure 5B). Principal component analysis (PCA) demonstrated that OE-3 line fruit samples separated clearly from WT fruit samples along PC1 or PC2 (Figure 7A), indicating differences in primary metabolites between OE-3 and WT. A total of 489 compounds were detected, among which 142 were identified to be altered significantly by *SlAAE3-1* overexpression (Supplementary Table 2). Analysis of the 142 compounds showed that most of them were detected at 20 DPA (67 compounds) and MG (75 compounds) stages, only a few of them were found at Pk (20 compounds) and RR (36 compounds) stages (Figure 7B). We observed that the majority of compounds that remarkably increased were amino acids, nucleotides, and their derivatives, while the compounds that decreased were mainly lipids (Figure 7C). It was clear that overexpression of *SlAAE3-1* improved carbon and nitrogen metabolisms. In addition, a total of 21 metabolites (14 phenolic acids, 3 amino acids and derivatives, 2 organic acids, 1 nucleotide and derivatives, and 1 other compound) were identified to be altered significantly, with a fold change more than 10 times (Table 1).

**TABLE 1:** Classification, fold change, regulation pattern, and development stages of compounds with more than 10-fold change.

Classification	Compounds	Fold change	Regulation pattern	Stage
Henolic acids	Pyrocatechol	438.91	Down	Pk
	p-Coumaraldehyde	736.95	Down	Pk
	p-Coumaryl alcohol	1240.67	Down	Pk
	3-*O*-p-Coumaroylshikimic acid	302.03	Down	20DPA
	3-*O*-p-Coumaroylshikimic acid	815.74	Down	MG
	Syringaldehyde-4-*O*-glucoside	951.74	Down	20DPA
	Benzyl-(2″-*O*-glucosyl)glucoside	1244.04	Down	20DPA
	4-*O*-(6′-*O*-Glucosylcaffeoyl)-4-hydroxybenzoic acid	371.14	Down	Pk
	6′-*O*-Feruloyl-D-sucrose	1589.33	Down	20DPA
	3,4,5-Tricaffeoylquinic acid	560.91	Down	MG
	Tyrosol	806.45	Up	MG
	2,6-Dihydroxybenzoic acid	1983.34	Up	20DPA
	Rhododendrol	11111.11	Up	RR
	Methyl 2,4-dihydroxyphenylacetate	1315.79	Up	20DPA
	Specnuezhenide	606.06	Up	20DPA
Organic acids	6-Hydroxyhexanoic acid	2338.48	Down	20DPA
	Mevalonic acid	17434.81	Down	MG
Nucleotides and derivatives	5-Methylcytosine	980.39	Up	20DPA
Amino acids and derivatives	γ-Glu-Cys	581.39	Up	20DPA
	L-Lysine-Butanoic acid	12.13	Up	20DPA
	Phenylacetyl-L-glutamine	934.58	Up	RR
Others	N-Acetyl-D-glucosamine-1-phosphate	5474.04	Down	RR

### Transcriptomics Profiles of Tomato at Different Maturation Stages

**FIGURE 8:**
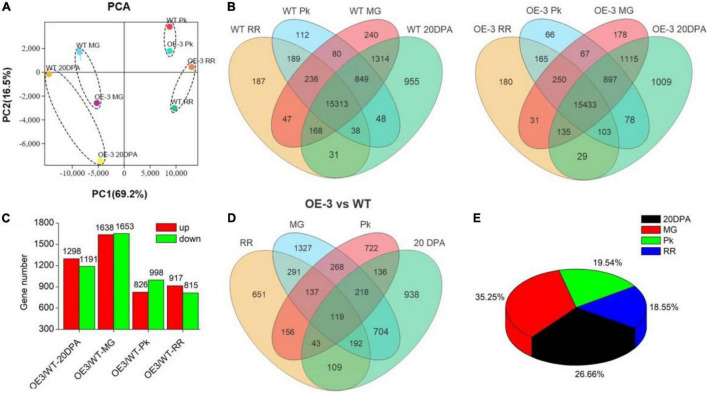
Identification of differentially expressed genes (DEGs) in Ailsa Craig tomato in OE-3. **(A)** Principal component analysis (PCA) of DEGs. PCA score plots (PC1 vs. PC2) for the first two principal components under SlAAE3-1 overexpression. Each sample was marked using individual color. **(B)** Venn diagrams showing overlap of all detected genes in WT and OE-3, respectively. **(C)** Statistics of DEGs in OE-3 compared with WT at different development stages. Overlap **(D)** and percentage occupied **(E)** by DEGs over development stages.

To get a further understanding about the effect of *SlAAE3-1* on fruit quality, the same samples used for metabolomics were subjected to RNA-Seq analysis. A total of 19,807 and 19,736 genes were identified in WT and OE-3 lines, respectively. We randomly selected 20 genes to check the accuracy of RNA-Seq by qRT-PCR and found 14 of them showed a similar expression tendency between qRT-PCR and RNA-Seq (Supplementary Figure 6). PCA revealed that OE-3 fruits separated clearly from WT along PC1 or PC2 (Figure 8A), indicating a difference in genes expression patterns between OE-3 and WT. However, they shared a similar distribution when divided by development stages (Figure 8B). Compared with WT, 2489 genes in 20DPA, 3291 genes in MG, 1824 genes in Pk, and 1732 genes in RR stages in OE-3 were considered as differentially expressed genes (DEGs) (Figure 8C). Most of these DEGs were detected in 20DPA and MG stages, up to 26.66 and 35.25%, respectively, and only 122 were differentially expressed throughout the development (Figures 8D,E). These data suggested that overexpression of *SlAAE3-1* mainly affected the early development of tomato fruit, which was in agreement with metabolomics.

**FIGURE 9:**
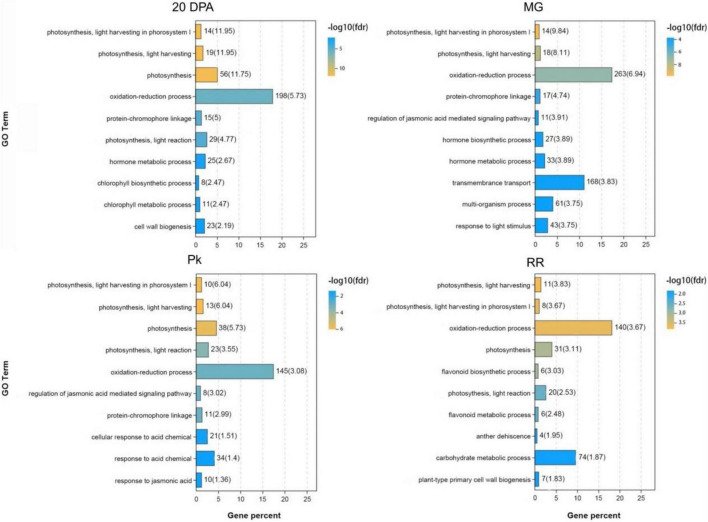
Gene ontology (GO) analysis of DEGs between WT and OE-3 at different development stages. The vertical axis indicates the GO Terms with –log10(fdr) ranking top 10. The horizontal axis indicates the gene percent among the DEGs.

Gene Ontology (GO) and Kyoto Encyclopedia of Genes and Genomes (KEGG) analysis of DEGs identified from comparisons between OE-3 and WT fruits provided more information about the effects of *SlAAE3* on fruit. At each development stage, DEGs related to the oxidation-reduction process constitutes the biggest group (Figure 9). Other DEGs were enriched in various biological pathways, including carbohydrate metabolism, amino acid metabolism, terpenoids and polyketides metabolism, lipid metabolism, nucleotide metabolism, biosynthesis of other secondary metabolites, signal transduction, and environmental adaptation (Supplementary Figure 7).

### Co-expression Analysis Identified Genes Accounting for Changes in Metabolites

**FIGURE 10:**
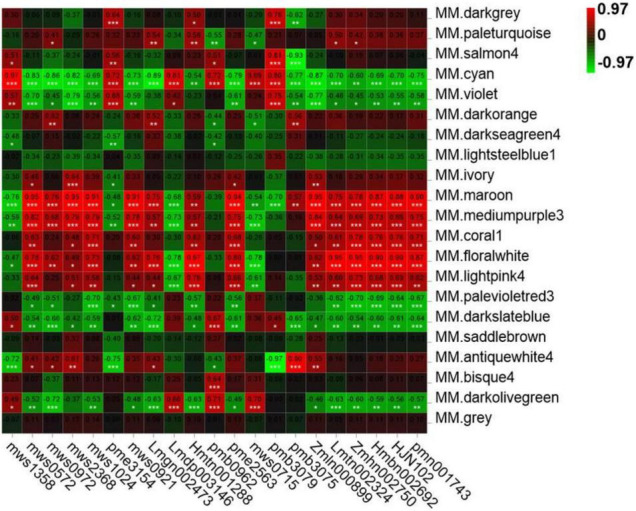
Module-trait associations based on Person correlations. The color key from green to red represents γ^2^ values from –0.97 to 0.97.

**FIGURE 11:**
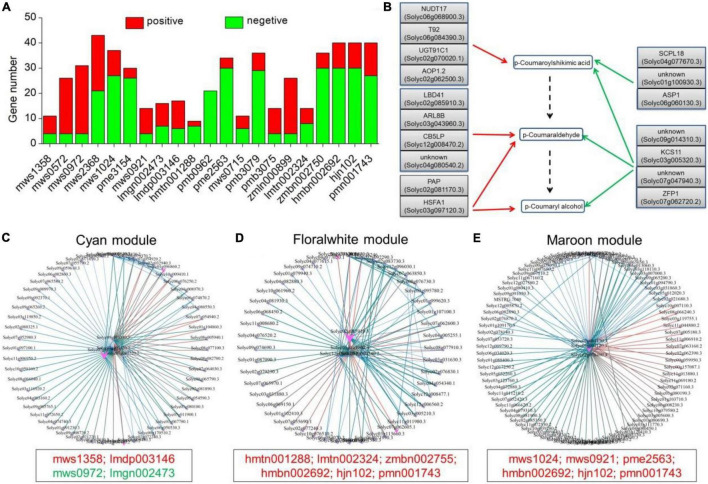
Key candidate genes and gene networks involved in part of the metabolites as identified by WGCNA. **(A)** Statistics of key candidate genes of metabolites with more than 10-fold change. **(B)** Key candidate genes involved in the regulation of p-Coumaroylshikimic acid, p-Coumaraldehyde, and p-Coumaryl alcohol. Red and green arrows represent positive and negative correlations, respectively. The dashed arrow represents the predicted metabolic pathway. **(C)** Gene network for the cyan module, which positively correlated with mws1358 (γ^2^ = 0.97), lmdp003146 (γ^2^ = 0.81), and negatively with mws0972 (γ^2^ = –0.86), lmgn002473 (γ^2^ = –0.89). **(D)** Gene network for the floralwhite module, which positively correlated with hmtn001288 (γ^2^ = 0.97), lmtn002324 (γ^2^ = 0.95), zmbn002750 (γ^2^ = 0.95), hmbn002692 (γ^2^ = 0.9), hjn102 (γ^2^ = 0.9), and pmn001743 (γ^2^ = 0.87). **(E)** Gene network for the maroon module, which positively correlated with mws1024 (γ^2^ = 0.91), mws0921 (γ^2^ = 0.91), pme2563 (γ^2^ = 0.94), hmbn002692 (γ^2^ = 0.87), hjn102 (γ^2^ = 0.88), and pmn001743 (γ^2^ = 0.9).

As shown in Table 1, a total of 21 metabolites displayed a fold change from 10 to 10000, which are clearly related to *SlAAE3-1*. We next carried out the weighted gene coexpression network analysis (WGCNA) for the transcriptome data using online tools provided by GENE *DENOVO*^®^.^11^ The metabolites listed in Table 1 were used as phenotypic data for WGCNA, and the transcriptome data were divided into 21 gene modules which were labeled with distinct colors. We carried out a correlation analysis between gene modules and phenotypic data and the result was displayed as a heat map (Figure 10). For every metabolite, the gene modules with a correlation coefficient (*r*^2^) higher than 0.9 were taken as the key modules, and hub genes having the most connections in the network were regarded as key genes in the regulation of the metabolites. We count the genes for each metabolite (Figure 11A). Interestingly, among the metabolites, we found three belonging to the coumarin derivatives: p-Coumaroylshikimic acid, p-Coumaraldehyde, and p-Coumaryl alcohol. The annotations for key genes are listed in Figure 11B. Four genes positively correlated with p-Coumaroylshikimic acid, 7 genes positively correlated with p-Coumaraldehyde, among which 4 showed a positive correlation with p-Coumaryl alcohol as well. At the same time, 7 genes negatively correlated with p-Coumaroylshikimic acid, among which 4 genes showed a negative correlation with both p-Coumaraldehyde and p-Coumaryl alcohol. In total, 18 genes correlated with coumarin derivatives above, among which 4 have not yet been annotated. We also found pyrocatechol (mws1358) and rhododendrol (lmdp003146) were positively correlated with cyan module, while 6-hydroxyhexanoic acid (mws0972) and 2,6-dihydroxybenzoic acid (lmgn002473) correlated negatively. P-Coumaraldehyde (mws1024), p-coumaryl alcohol (mws0921), and γ-glu-cys (pme2563) positively correlated with maroon module, methyl 2,4-dihydroxyphenylacetate (hmtn001288), benzyl-(2″-*O*-glucosyl)glucoside (lmtn002324), and 4-*O*-(6′-*O*-Glucosylcaffeoyl)-4-hydroxybenzoic acid (zmbn002750) positively correlated with floralwhite module, while 6′-*O*-feruloyl-D-sucrose (hmbn002692),3,4,5-tricaffeoylquinic acid (hjn102) and specnuezhenide (pmn001743) were positively correlated with both maroon and floralwhite modules (Figures 11C–E).

## Discussion

### The Relationship Between SlAAE3-1 and SlAAE3-2 Proteins

The amino acid sequences of SlAAE3-1 and SlAAE3-2 share a similarity of 82%, and their main difference locates between 1–60 and 120–150 amino acids. Despite these differences in amino acids, both SlAAE3-1 and SlAAE3-2 localized to the cytoplasm, which coincides with its role in the acetylation degradation pathway of oxalic acid (Kubicek et al., 1988). However, under the same condition, SlAAE3-1 displayed a higher affinity toward oxalate compared with SlAAE3-2. In fact, we failed to measure the enzymatic activity of SlAAE3-2. Since the AAE3 in *Arabidopsis thaliana* (Foster et al., 2012), *Medicago truncatula* (Foster et al., 2016), and *Oryza sativa* (Liu et al., 2016; Peng et al., 2017) also displayed a different enzymatic activity, we propose the possibility that the AMP-binding domain and acetyl-CoA synthetase domain determine the function of oxalate consumption of AAE3, while their enzymatic activity is determined by the regions between 1–60 and 120–150 amino acids. On the other hand, SlAAE3-2 showed a much stronger expressing level compared with SlAAE3-1 in flowers, indicating that they have distinct roles in different tissues. Liu et al. (2016) found the overexpression of *OsAAE3* repressed the floret development. Therefore, it is possible that SlAAE3-2 also participate in the development and metabolism of tomato flower.

Interestingly, we found that the expression level of *SlAAE3-1* and *SlAAE3-2* decreased in the leaves when detached from the plant, but they were induced by oxalate both in WT and mutant lines. Moreover, *SlAAE3-2* showed a higher expression induction than *SlAAE3-1* by oxalate treatment. Therefore, the expressions of *SlAAE3-1* and *SlAAE3-2* were also regulated by signals or metabolites from other parts of the plant, and there were some differences in their induction pattern by oxalate. Meanwhile, the expression of *SlAAE3-2* decreased in mutant lines compared to WT, probably because the normal metabolism was destroyed by oxalate in the mutant lines missing *SlAAE3-1*.

### Knocking Out of SlAAE3-1 Improves Tomato Fruit Quality

There are many studies about the impacts of oxalate on fruit or seed nutrient quality, in which sugar-acid ratio is frequently used as a key index of fruit quality (Chakraborty et al., 2013; Kumar et al., 2016; Joshi et al., 2021). In tomato, glucose and fructose are two major sugars (Paponov et al., 2019), and malate and citrate are the main acids (Azzi et al., 2015). To explore the impacts of *SlAAE3-1* on fruit quality, we measured the contents of main sugars and acids from the fruits of WT and mutant lines. The results showed that loss-of-function mutation of *SlAAE3-1* resulted in the increase in the contents of oxalate, glucose, fructose, and sucrose, whereas the contents of citrate and malate decreased. These changes finally led to a higher sugar-acid ratio in the mutant lines (Figure 6). Therefore, using sugar-acid ratio as indicator, we proposed that function impairment of *SlAAE3-1* improves the fruit quality of MT. In contrast, Joshi et al. (2021) reported that the decreased oxalate content improves spinach food quality. However, spinach food quality was evaluated by the dietary bioavailability of calcium and other minerals, ascorbates, and vitamins rather than the sugar-acid ratio. Tang carried out gas chromatography-mass spectrometry (GC-MS) and transcriptome analysis on a cherry tomato and found fruit quality deterioration went along with a decrease in organic acids, which was regarded as the reason for fruit quality deterioration (Tang et al., 2020). Which obviously lacks other parameters to draw such a conclusion.

Mineral nutrient content is another index to evaluate fruit quality. We measured the content of nine mineral nutrients at different fruit development stages of WT and mutant lines (Supplementary Figure 4). At the RR stage, Na, Mg, Fe, Zn, Ca, P, and Mn displayed a higher content in the mutant lines compared with WT, which also proved the absence of SlAAE3-1 improves the fruit quality of MT. Although oxalate can affect mineral content, the results of different studies are a bit contradictory. Kumar et al. overexpressed an oxalate decarboxylase cloned from *Flammulina velutipes* in soya bean and grass pea and found that the reduction in oxalate content resulted in increased levels of some micronutrients (Kumar et al., 2016). On the contrary, Nakata held the opinion that due to the acidity and chelation properties of oxalate, plants with a high amount of oxalate need to store more mineral elements to maintain a stable pH environment in the cells. So, a positive correlation should be displayed between the contents of mineral elements and oxalate (Nakata, 2003). In our research, as the function of SlAAE3-1 is disabled, the content of sugars (mainly glucose and fructose) and organic acids (mainly malate and citrate) showed distinct changes, and their combined action resulted in altered contents of mineral elements.

### SlAAE3-1 Is Involved in Redox Metabolism

SlAAE3, an oxalyl-CoA synthetase, catalyzes the conversion of oxalate and CoA into oxalyl-CoA, which is not related to the redox process. However, Liu et al. (2016) found the activity of peroxidase decreased in OsAAE3 overexpression lines. Chemical modification and site-directed mutagenesis analysis identified thiols as the active site residues for OsAAE3 catalysis (Peng et al., 2017). These results suggest that OsAAE3 might be related to redox metabolism and the redox state itself. Our data showed that overexpressing SlAAE3-1 resulted in more than 10-fold change of 21 different metabolites. Among those compounds, 14 (nearly 60%) belong to phenolic acids, which are considered to have a reducing ability. GO analysis of DEGs showed most of them were classified into redox metabolism (Figure 9). Although there were no significant differences in the fruit phenotype between WT and OE-3 (Supplementary Figure 5B), the DEGs in WT were classified into carbohydrate metabolic and protein phosphorylation processes, while in OE-3 they were classified into oxidation-reduction and carbohydrate metabolic processes (Supplementary Figure 8). Therefore, it is likely that SlAAE3 plays a central role in regulating redox possibly *via* oxalate metabolism.

In conclusion, SlAAE3-1 functions as an oxalyl-CoA synthetase, driving the acetylation pathway of oxalate degradation, which affects the transcriptional and metabolic changes to control fruit quality.

## Data Availability Statement

The datasets presented in this study can be found in online repositories. The names of the repository/repositories and accession number(s) can be found in the article/Supplementary Material.

## Author Contributions

PL, QH, and JJ performed the experiment. YW helped to culture the plants. PL, YL, and GM analyzed the data. KZ cultured the plants and sampled the plants. PL wrote the manuscript. WF and JY designed the experiments and revised the final manuscript. All authors contributed to the article and approved the submitted version.

## Conflict of Interest

The authors declare that the research was conducted in the absence of any commercial or financial relationships that could be construed as a potential conflict of interest.

## Publisher’s Note

All claims expressed in this article are solely those of the authors and do not necessarily represent those of their affiliated organizations, or those of the publisher, the editors and the reviewers. Any product that may be evaluated in this article, or claim that may be made by its manufacturer, is not guaranteed or endorsed by the publisher.

## Footnotes

1
http://biogle.cn/

2
http://www.geneontology.org/

3
https://www.metware.cn/

4
http://www.anpel.com.cn/

5
www.shimadzu.com.cn/

6
www.appliedbiosystems.com.cn/

7
www.r-project.org

8
http://www.kegg.jp/kegg/compound/

9
http://www.kegg.jp/kegg/pathway.html

10
https://phytozome.jgi.doe.gov/pz/portal.html#

11
https://www.omicsmart.com/
