# Supplementary material for: Tomato Oxalyl-CoA Synthetase Degrades Oxalate and Affects Fruit Quality
Source: Front Plant Sci. 2022 Jul 7;13:951386. doi: 10.3389/fpls.2022.951386 (PMC9301600; doi:10.3389/fpls.2022.951386)
Supplement: Supplementary file 1 [file Data_Sheet_1.docx]

**Supplemental Table S1.** Sequence information of primers. F, forward primer. R, reverse primer.

| **Gene** | **Use** | **Sequence(5' to 3')** |
| --- | --- | --- |
| SlAAE3-1 | recombinant protein expression | F: cagcaaatgggtcgcggatccATGGAGAGTATGACGCTCACTGG |
|  |  | R: tgcggccgcaagcttgtcgacCTACGCTCCAAATTTAGGGACTTT |
|  | qPCR | F: TCATACATCAGGCACCACGAG |
|  |  | R: GCAGATGGAAGTGTCACGGA |
|  | subcellular localization construct | F: gagaacacgggggacgagctcATGGAGAGTATGACGCTCACTGG |
|  |  | R: ttcttctcctttactgtcgacCGCTCCAAATTTAGGGACTTTG |
|  | DNA lesions identified | F: GCTGTAATTCGAGTTAGAGCC |
|  |  | R: TACCAAATTAAGCTGAGAAAGAG |
|  | Overexpression lines | F: gagaacacgggggacgagctcATGGAGAGTATGACGCTCACTGG |
|  |  | R: ttcttctcctttactgtcgacCTACGCTCCAAATTTAGGGACTTT |
| SlAAE3-2 | recombinant protein expression | F: cagcaaatgggtcgcggatccATGGCAACGCTCACAATGTTATT |
|  |  | R: tgcggccgcaagcttgtcgacTCAAGCTCCAAACTTAGGCACTT |
|  | subcellular localization construct | F: gagaacacgggggacgagctcATGGCAACGCTCACAATGTTATT |
|  |  | R: ttcttctcctttactgtcgacAGCTCCAAACTTAGGCACTTTAGC |
| Actin | qPCR and DNA lesions identified | F: AGGCAGGATTTGCTGGTGATGATGCT |
|  |  | R: ATACGCATCCTTCTGTCCCATTCCGA |
| solyc06g068900.3 | qPCR | F: CCTAAGGGTGGTTGGGAGAT |
|  |  | R: CATAAACGACGACGAGCAGAT |
| solyc03g005320.3 |  | F: AAGGTCGGGTTTAGGTCAAA |
|  |  | R: CACCATCTCAGCCTCTTTCC |
| solyc09g014310.3 |  | F: TGGGCAGCATTACTAGACCTT |
|  |  | R: GGATGGGATTCATACCAACAT |
| solyc01g067560.3 |  | F: AATCAAATCCCGGACTATGG |
|  |  | R: CTCCTCAATCTTCTCGGTGG |
| solyc03g043960.3 |  | F: ATGGGTCTTTGGGATGCTT |
|  |  | R: TACTGCGGAATCTGGGTTG |
| solyc02g085910.3 |  | F: GTAAACAACAACGGTCCTCCT |
|  |  | R: TCAGACTGGTGACTCAACGAA |
| solyc09g083090.3 |  | F: ATTGGTTCGTCTGTTGGGTC |
|  |  | R: AGGATATTCGGGTGGTGATT |
| solyc04g077670.3 |  | F: ATGATTCCATTTCTTGTTGGC |
|  |  | R: TTGGCTGCATTGAGCATTAG |
| solyc02g083670.3 |  | F: GCCGAAGATGGATGGTTATG |
|  |  | R: AGTCAAGGAAAGAATGGTGGA |
| solyc01g081010.3 |  | F: TGTTGGAAGCCAGAGCGA |
|  |  | R: GCAGAATAAACACCAGCACCT |
| solyc02g085600.1 |  | F: AGCGTAGGATTTGATTATGTGG |
|  |  | R: CGTCGGAGGAACTTGAAGAA |
| solyc04g080290.3 |  | F: GCGGAACTGATGAAGTCGTAC |
|  |  | R: TGAGCCATTTGCCTTGAGA |
| solyc01g086650.3 |  | F: CGGCTGACCCTGACTCTACA |
|  |  | R: GCTACCACCTTCCCAATGAT |
| solyc07g018010.3 |  | F: TGTGGTCCCAAACATAAGCA |
|  |  | R: TTCTTTGACGATGATCTTCCAG |

**Supplemental Table S2.** Index, classification, and formula of compounds significational induced by SlAAE3-1 overexpression.

| **Index** | **Compounds** | **Class** | **Formula** |
| --- | --- | --- | --- |
| mws1358 | Pyrocatechol | Phenolic acids | C6H6O2 |
| mws1336 | 4-Aminobenzoic acid | Phenolic acids | C7H7NO2 |
| lmgn001670 | Salicylic acid | Phenolic acids | C7H6O3 |
| mws2368 | Tyrosol | Phenolic acids | C8H10O2 |
| mws1024 | p-Coumaraldehyde | Phenolic acids | C9H8O2 |
| mws2213 | Cinnamic acid | Phenolic acids | C9H8O2 |
| mws0921 | p-Coumaryl alcohol | Phenolic acids | C9H10O2 |
| lmgn002473 | 2,6-Dihydroxybenzoic acid | Phenolic acids | C7H6O4 |
| pmb0142 | Caffeic aldehyde | Phenolic acids | C9H8O3 |
| mws0467 | 3-(4-Hydroxyphenyl)-propionic acid | Phenolic acids | C9H10O3 |
| lmdp003146 | Rhododendrol | Phenolic acids | C10H14O2 |
| hmtn001288 | Methyl 2,4-dihydroxyphenylacetate | Phenolic acids | C9H10O4 |
| mws0014 | Ferulic acid | Phenolic acids | C10H10O4 |
| pme3443 | Sinapinaldehyde | Phenolic acids | C11H12O4 |
| mws1212 | Methyl ferulate | Phenolic acids | C11H12O4 |
| mws0853 | Sinapyl alcohol | Phenolic acids | C11H14O4 |
| mws1521 | Salicin | Phenolic acids | C13H18O7 |
| pmb3142 | Salicylic acid-2-O-glucoside* | Phenolic acids | C13H16O8 |
| zmhn000892 | 4-O-Glucosyl-3,4-dihydroxybenzyl alcohol | Phenolic acids | C13H18O8 |
| pmb3075 | 3-O-p-Coumaroylshikimic acid* | Phenolic acids | C16H16O7 |
| pmb0751 | Trans-5-O-(p-Coumaroyl)shikimate* | Phenolic acids | C16H16O7 |
| zmhn002301 | p-Coumaric acid-4-O-glucoside | Phenolic acids | C15H18O8 |
| pmn001419 | 1-O-[(E)-p-Cumaroyl]-D-glucose | Phenolic acids | C15H18O8 |
| pmn001690 | 3-Hydroxy-4-isopropylbenzylalcohol-3-O-glucoside | Phenolic acids | C16H24O7 |
| hmln002806 | 5-O-Caffeoylshikimic acid | Phenolic acids | C16H16O8 |
| pmn001421 | 3-O-p-Coumaroylquinic acid* | Phenolic acids | C16H18O8 |
| pma6460 | 4-O-p-Coumaroylquinic acid* | Phenolic acids | C16H18O8 |
| pmb3074 | 5-O-p-Coumaroylquinic acid* | Phenolic acids | C16H18O8 |
| mws0906 | Coniferin | Phenolic acids | C16H22O8 |
| lmmn000774 | Dihydrocaffeoylglucose | Phenolic acids | C15H20O9 |
| zmln000899 | Syringaldehyde-4-O-glucoside | Phenolic acids | C15H20O9 |
| pme1816 | Neochlorogenic acid (5-O-Caffeoylquinic acid)* | Phenolic acids | C16H18O9 |
| zmhn002334 | 6-O-Feruloyl-D-glucose* | Phenolic acids | C16H20O9 |
| pma3724 | 1-O-Feruloylquinic acid* | Phenolic acids | C17H20O9 |
| mws0179 | Chlorogenic acid methyl ester | Phenolic acids | C17H20O9 |
| pmb0752 | 3-O-Feruloylquinic acid* | Phenolic acids | C17H20O9 |
| lmgn003073 | 5-O-Feruloylquinic acid* | Phenolic acids | C17H20O9 |
| pmn001695 | Trihydroxycinnamoylquinic acid | Phenolic acids | C16H20O10 |
| lmzn001582 | 5'-Glucosyloxyjasmanic acid | Phenolic acids | C18H28O9 |
| lmtn002324 | Benzyl-(2''-O-glucosyl)glucoside | Phenolic acids | C19H28O11 |
| cmzn005251 | 6-O-Caffeoylarbutin | Phenolic acids | C21H22O10 |
| pmb2654 | Anthranilate-1-O-Sophoroside | Phenolic acids | C19H27NO12 |
| zmhn002750 | 4-O-(6'-O-Glucosylcaffeoyl)-4-hydroxybenzoic acid | Phenolic acids | C22H22O11 |
| zmhn002508 | 4-p-Cumaroyl-rhamnosyl-(1→6)-D-glucose | Phenolic acids | C21H28O12 |
| pmn001707 | Quillaic acid | Phenolic acids | C30H46O5 |
| hmcn001296 | Cistanoside F | Phenolic acids | C21H28O13 |
| li512115 | Isochlorogenic acid B | Phenolic acids | C25H24O12 |
| hmbn002692 | 6'-O-Feruloyl-D-sucrose | Phenolic acids | C22H30O14 |
| hjn102 | 3,4,5-Tricaffeoylquinic acid | Phenolic acids | C34H30O15 |
| pmn001743 | Specnuezhenide | Phenolic acids | C31H42O17 |
| pma0134 | D-(-)-Threose | Others | C4H8O4 |
| pme0490 | Nicotinic acid (Vitamin B3) | Others | C6H5NO2 |
| pme2529 | 1,5-Anhydro-D-glucitol | Others | C6H12O5 |
| ma10039492 | Dehydroascorbic acid | Others | C6H6O6 |
| mws1155 | D-Mannitol | Others | C6H14O6 |
| zmpn000199 | D-Galactaric acid | Others | C6H10O8 |
| hmcn000192 | Sedoheptulose | Others | C7H14O7 |
| pma6455 | Ribulose-5-phosphate | Others | C5H11O8P |
| pme3313 | D-Fructose 6-phosphate | Others | C6H13O9P |
| pme3163 | D-Sedoheptuiose 7-phosphate | Others | C7H15O10P |
| pmb3079 | N-Acetyl-D-glucosamine-1-phosphate | Others | C8H16NO9P |
| lmqn000432 | 1-(sn-Glycero-3-phospho)-1D-myo-inositol | Others | C9H19O11P |
| pme2125 | Raffinose | Others | C18H32O16 |
| mws1589 | D-Panose | Others | C18H32O16 |
| pmb2653 | D(+)-Melezitose O-rhamnoside | Others | C24H42O20 |
| mws4052 | 1-Aminocyclopropane-1-carboxylic acid | Organic acids | C4H7NO2 |
| pme1216 | 2-Picolinic acid | Organic acids | C6H5NO2 |
| lmbp000668 | Isonicotinic acid | Organic acids | C6H5NO2 |
| mws0236 | 2-Aminoethanesulfonic acid | Organic acids | C2H7NO3S |
| mws0425 | Citraconic acid | Organic acids | C5H6O4 |
| pme0274 | 6-Aminocaproic acid | Organic acids | C6H13NO2 |
| lmgn000160 | 3-Ureidopropionic Acid | Organic acids | C4H8N2O3 |
| mws0341 | 2-Hydroxyisocaproic acid | Organic acids | C6H12O3 |
| lmrn002746 | 2-Hydroxy-4-methylpentanoic acid | Organic acids | C6H12O3 |
| mws0972 | 6-Hydroxyhexanoic acid | Organic acids | C6H12O3 |
| pme3154 | Mevalonic acid | Organic acids | C6H12O4 |
| mws0262 | L-Tartaric acid | Organic acids | C4H6O6 |
| mws0154 | Shikimic acid | Organic acids | C7H10O5 |
| pme1654 | Jasmonic acid | Organic acids | C12H18O3 |
| pme2074 | (-)-Jasmonoyl-L-Isoleucine | Organic acids | C18H29NO4 |
| mws0572 | 5-Methylcytosine | Nucleotides and derivatives | C5H7N3O |
| pme0040 | Adenine | Nucleotides and derivatives | C5H5N5 |
| pme1173 | Allopurinol | Nucleotides and derivatives | C5H4N4O |
| pme0256 | Xanthine | Nucleotides and derivatives | C5H4N4O2 |
| pmc0274 | 6-Methylmercaptopurine | Nucleotides and derivatives | C6H6N4S |
| pmb2507 | 2-Deoxyribose-1-phosphate | Nucleotides and derivatives | C5H11O7P |
| pme1194 | 2'-Deoxycytidine | Nucleotides and derivatives | C9H13N3O4 |
| pme3732 | Cytidine | Nucleotides and derivatives | C9H13N3O5 |
| ml1018052 | Cytarabine | Nucleotides and derivatives | C9H13N3O5 |
| mws0248 | Uridine | Nucleotides and derivatives | C9H12N2O6 |
| mws1060 | 9-(Arabinosyl)hypoxanthine | Nucleotides and derivatives | C10H12N4O5 |
| pme3174 | Cytidine 5'-monophosphate(Cytidylic acid) | Nucleotides and derivatives | C9H14N3O8P |
| pmc0066 | 2'-Deoxyinosine-5'-monophosphate | Nucleotides and derivatives | C10H13N4O7P |
| pmb2640 | Dodecanoic acid (Lauric acid) | Lipids | C12H24O2 |
| mws0361 | Palmitoleic Acid | Lipids | C16H30O2 |
| mws0120 | Choline Alfoscerate | Lipids | C8H20NO6P |
| zmyn004714 | Ricinoleic acid | Lipids | C18H34O3 |
| pmf0297 | 1-Eicosanol | Lipids | C20H42O |
| pmb2789 | 13S-Hydroperoxy-6Z,9Z,11E-octadecatrienoic acid | Lipids | C18H30O4 |
| pmn001691 | 9,12,13-Trihydroxy-10,15-octadecadienoic acid | Lipids | C18H32O5 |
| lmhp008337 | LysoPE 14:0(2n isomer) | Lipids | C19H40NO7P |
| pmb0876 | LysoPE 16:0 | Lipids | C21H44NO7P |
| pmd0160 | LysoPE 16:0(2n isomer) | Lipids | C21H44NO7P |
| pmb0881 | LysoPE 18:2 | Lipids | C23H44NO7P |
| mws0289 | LysoPE 18:1 | Lipids | C23H46NO7P |
| pmd0132 | LysoPC 16:0(2n isomer) | Lipids | C24H50NO7P |
| hmqp006235 | LysoPC 18:4 | Lipids | C26H46NO7P |
| pmd0136 | LysoPC 18:0(2n isomer) | Lipids | C26H54NO7P |
| lmhp010908 | LysoPC 19:1 | Lipids | C27H54NO7P |
| lmhp009890 | LysoPC 20:3 | Lipids | C28H52NO7P |
| lmxp010913 | 1-(9Z-Octadecenoyl)-2-(9-oxo-nonanoyl)-sn-glycero-3-phosphocholine | Lipids | C35H66NO9P |
| pmb0168 | PC 16:1/14:1 | Lipids | C38H70NO8P |
| pme0006 | L-Proline | Amino acids and derivatives | C5H9NO2 |
| mws0256 | L-Valine | Amino acids and derivatives | C5H11NO2 |
| mws0230 | L-Threonine | Amino acids and derivatives | C4H9NO3 |
| mws0263 | 5-Oxo-L-Proline | Amino acids and derivatives | C5H7NO3 |
| ml10181668 | Cycloleucine | Amino acids and derivatives | C6H11NO2 |
| rfmb319 | Pipecolic acid | Amino acids and derivatives | C6H11NO2 |
| mws1587 | L-Norleucine | Amino acids and derivatives | C6H13NO2 |
| mws0258 | L-Isoleucine | Amino acids and derivatives | C6H13NO2 |
| mws0227 | L-Leucine | Amino acids and derivatives | C6H13NO2 |
| mws0219 | L-Aspartic Acid | Amino acids and derivatives | C4H7NO4 |
| pme1002 | L-Tyramine | Amino acids and derivatives | C8H11NO |
| pme0193 | L-Glutamine | Amino acids and derivatives | C5H10N2O3 |
| pme0026 | L-Lysine | Amino acids and derivatives | C6H14N2O2 |
| mws0254 | L-Histidine | Amino acids and derivatives | C6H9N3O2 |
| mws1550 | S-Allyl-L-cysteine | Amino acids and derivatives | C6H11NO2S |
| lmbp000123 | L-Homomethionine | Amino acids and derivatives | C6H13NO2S |
| pme0021 | L-Phenylalanine | Amino acids and derivatives | C9H11NO2 |
| mws1401 | L-Theanine | Amino acids and derivatives | C7H14N2O3 |
| zmyn000155 | N-α-Acetyl-L-ornithine | Amino acids and derivatives | C7H14N2O3 |
| mws0260 | L-Arginine | Amino acids and derivatives | C6H14N4O2 |
| mws0250 | L-Tyrosine | Amino acids and derivatives | C9H11NO3 |
| pme2602 | O-Phospho-L-serine | Amino acids and derivatives | C3H8NO6P |
| zmjp000182 | N-Monomethyl-L-arginine | Amino acids and derivatives | C7H16N4O2 |
| mws0282 | L-Tryptophan | Amino acids and derivatives | C11H12N2O2 |
| pmb0962 | L-Lysine-Butanoic Acid | Amino acids and derivatives | C10H22N2O4 |
| pme2563 | γ-Glu-Cys | Amino acids and derivatives | C8H14N2O5S |
| mws0715 | Phenylacetyl-L-glutamine | Amino acids and derivatives | C13H16N2O4 |
| mws1375 | Nicotianamine | Amino acids and derivatives | C12H21N3O6 |
| pme1086 | Glutathione reduced form | Amino acids and derivatives | C10H17N3O6S |
| pmb0464 | L-Aspartic acid-O-diglucoside | Amino acids and derivatives | C16H27NO14 |
