# Supplementary material for: Tomato Oxalyl-CoA Synthetase Degrades Oxalate and Affects Fruit Quality
Source: Front Plant Sci. 2022 Jul 7;13:951386. doi: 10.3389/fpls.2022.951386 (PMC9301600; doi:10.3389/fpls.2022.951386)
Supplement: Supplementary file 2 [file Data_Sheet_2.docx]

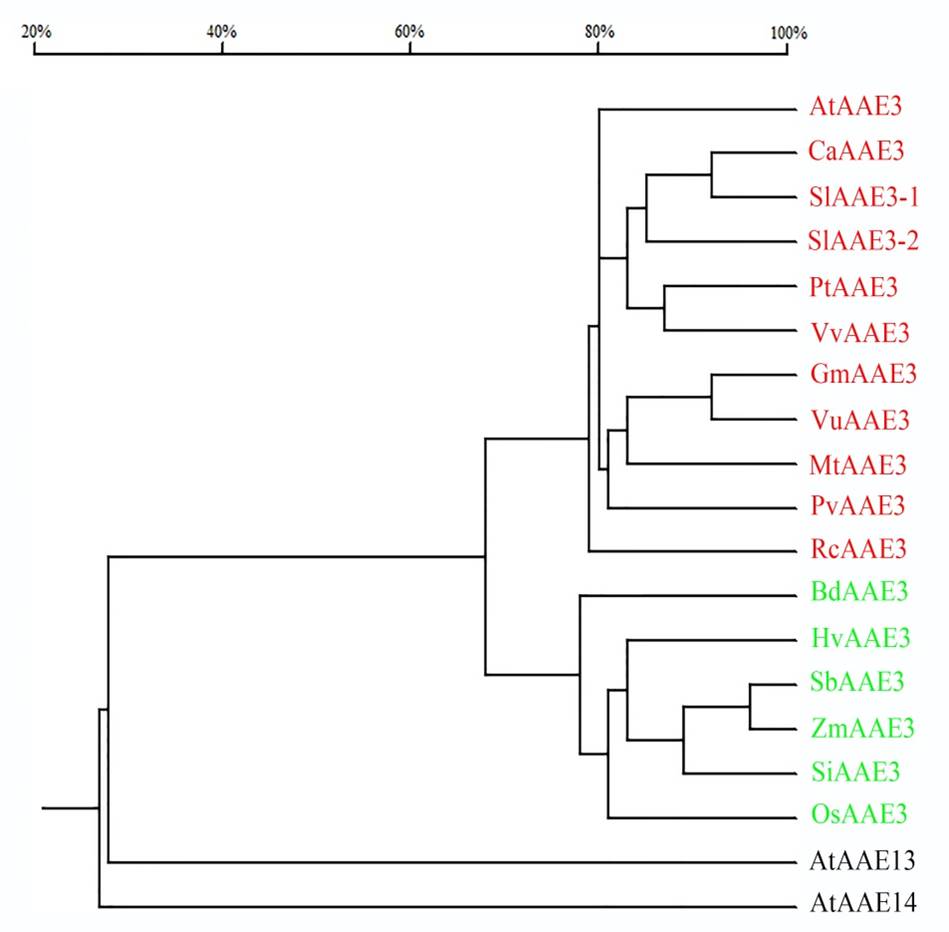


**Supplemental Figure S1.** Phylogram of AAE3 and two orthologous proteins in other plant species. AAE3 proteins derived from *Arabidopsis thaliana* (TAIR: AT3G48990), *Capsicum annuum* (GenBank:NP_001311686.1), *Solanum lycopersicum* (GenBank: XP_004234395.1,SlAAE3-1; XP_004240733.1,SlAAE3-2), *Populus trichocarpa* (GenBank: XP_002322473.1), *Vitis vinifera* (GenBank: XP_002267459.1), *Glycine max* (GenBank: XP_003534000.1), *Vigna umbellate* (GenBank:KX354978), *Medicago truncatula* (GenBank: XP_003599555.1), *Phaseolusn vulgaris* (GenBank: XP_007143422.1), *Ricinus communis* (GenBank: XP_002509782.2), *Brachypodium distachyon* (GenBank: XP_003579506.1), *Hordeum vulgare* (GenBank: BAK00674.1), *Sorghum bicolor* (GenBank: KXG27467.1), *Zea mays* (GenBank:AEY64280.1), *Setaria italic* (GenBank: XP_004960018.1), *Oryza sativa* (RAP: Os04g0683700). AtAAE13 (TAIR: AT3G16170) and AtAAE14 (TAIR: AT1G30520) which belong to superfamily VII of Aceyl-Activatig Enzymes were included in phylogram with AtAAE3.


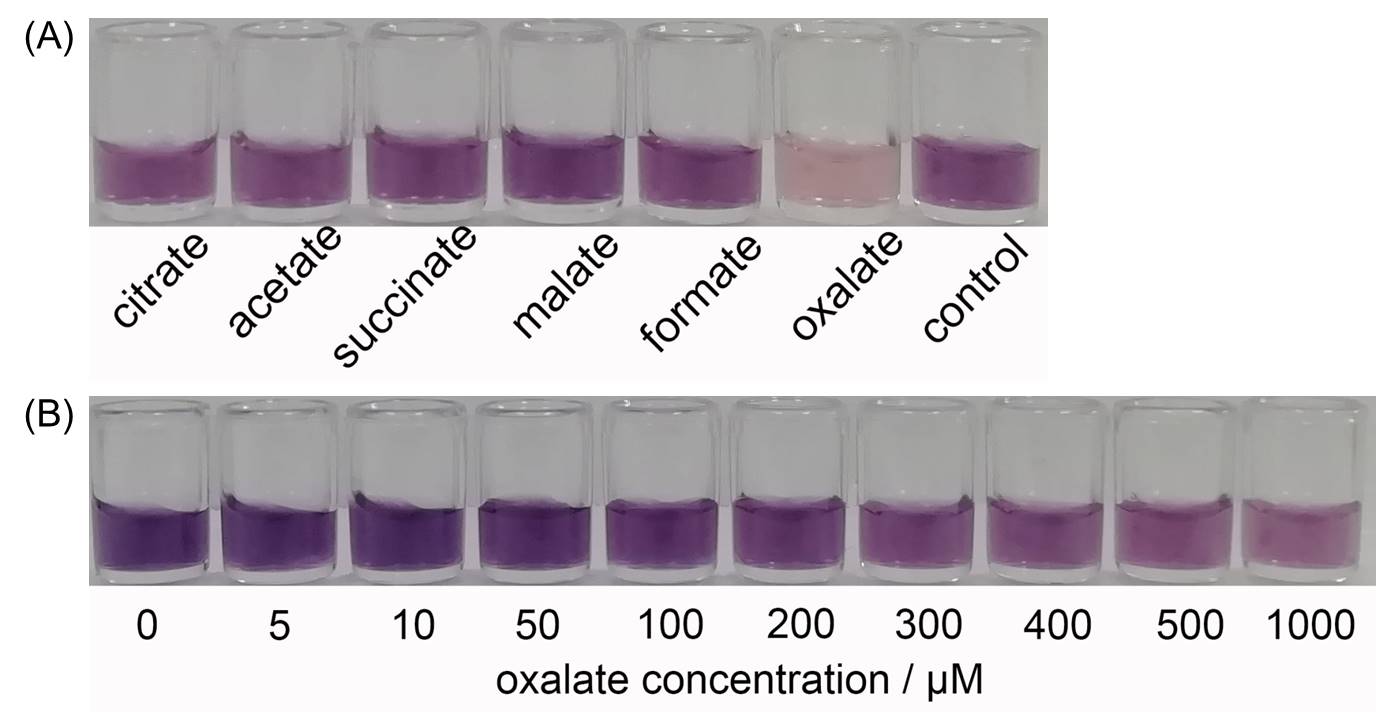


**Supplemental Figure S2.** Biochemical analysis of SlAAE3-2. (A) Reaction activity of SlAAE3-2 against different organic acids. NADH residue level indicated by nitroblue tetrazolium and 1-methoxy-5-methylphenazinium methosulfate. (B) Visual inspection of NADH residue level indicated by nitroblue tetrazolium and 1-Methooxy-5-methylphenazinium methosulfate. The reaction assay consumed more NADH as oxalate increased.


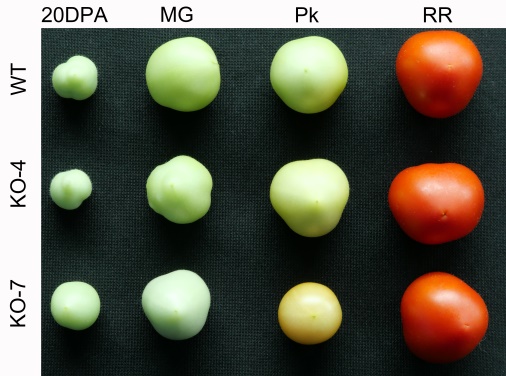


**Supplemental Figure S3.** Phenotypes of *SlAAE3-1* knock out mutant and WT fruits at different development stages.


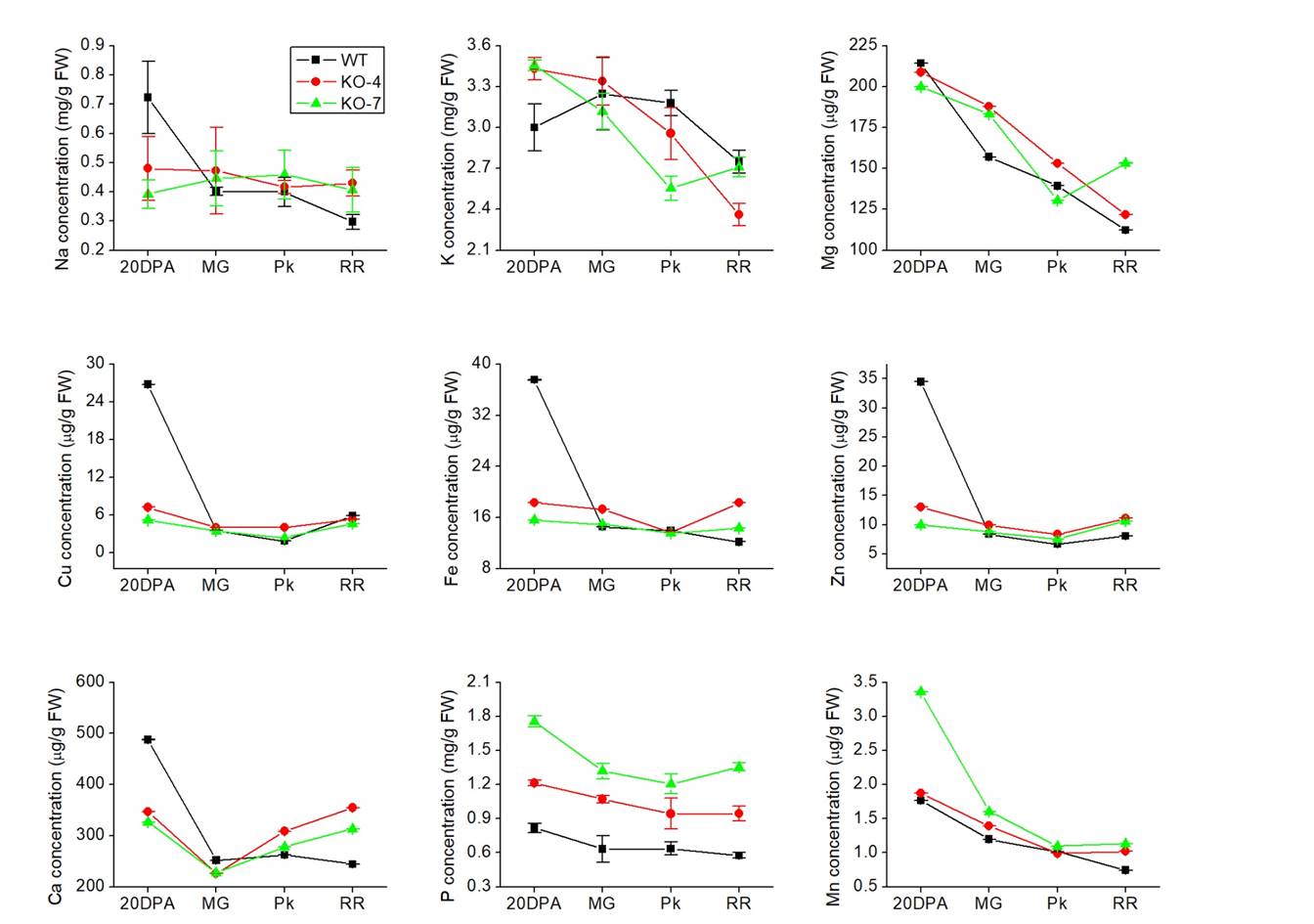


**Supplemental Figure S4.** The content of mineral elements in the fruits of WT, KO-4, and KO-7 lines at different development stages.


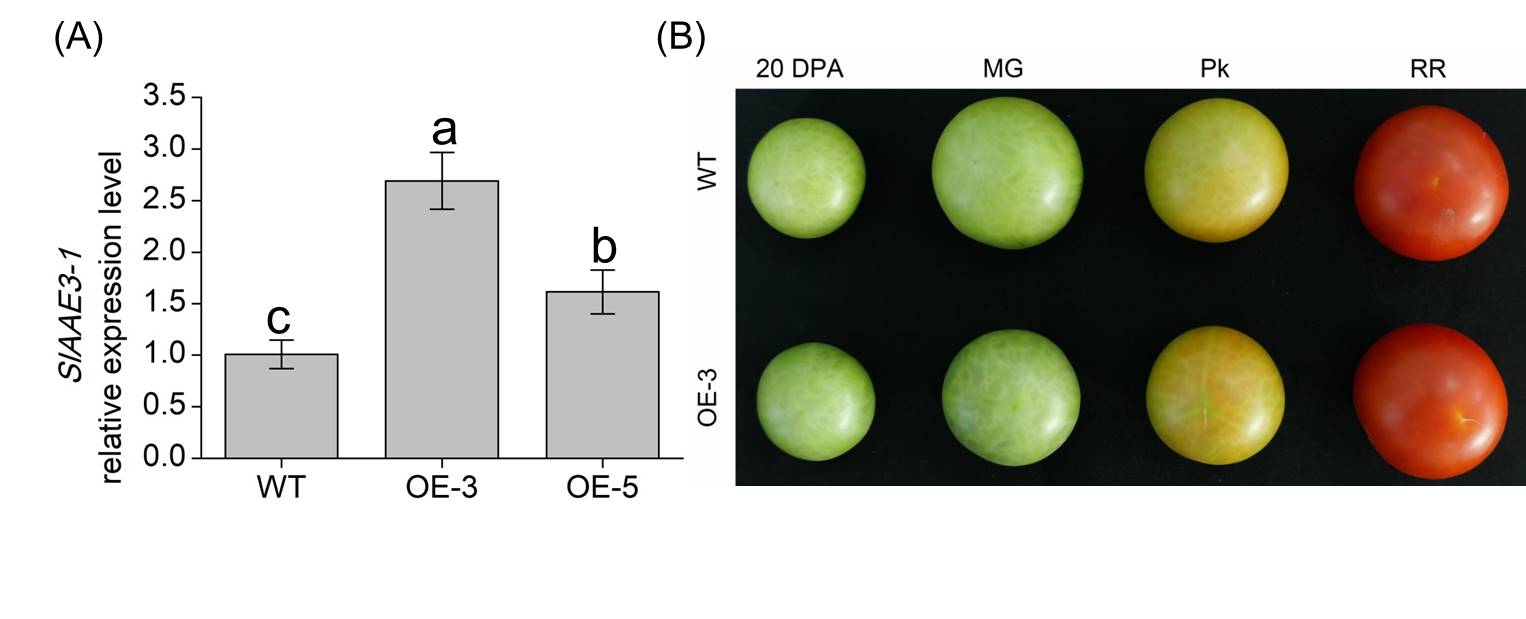


**Supplemental Figure S5.** SlAAE3-1 overexpression lines. (A) The relative expression level of SlAAE3-1 in OE-3 and OE-5 lines. (B) Phenotypes of OE-3 and WT fruits at different development stages used for transcriptome and metabolome. Error bars indicate mean values ± SD (n=3). Different letters on vertical bars indicate significant difference at *p* < 0.05 using one-way ANOVA.


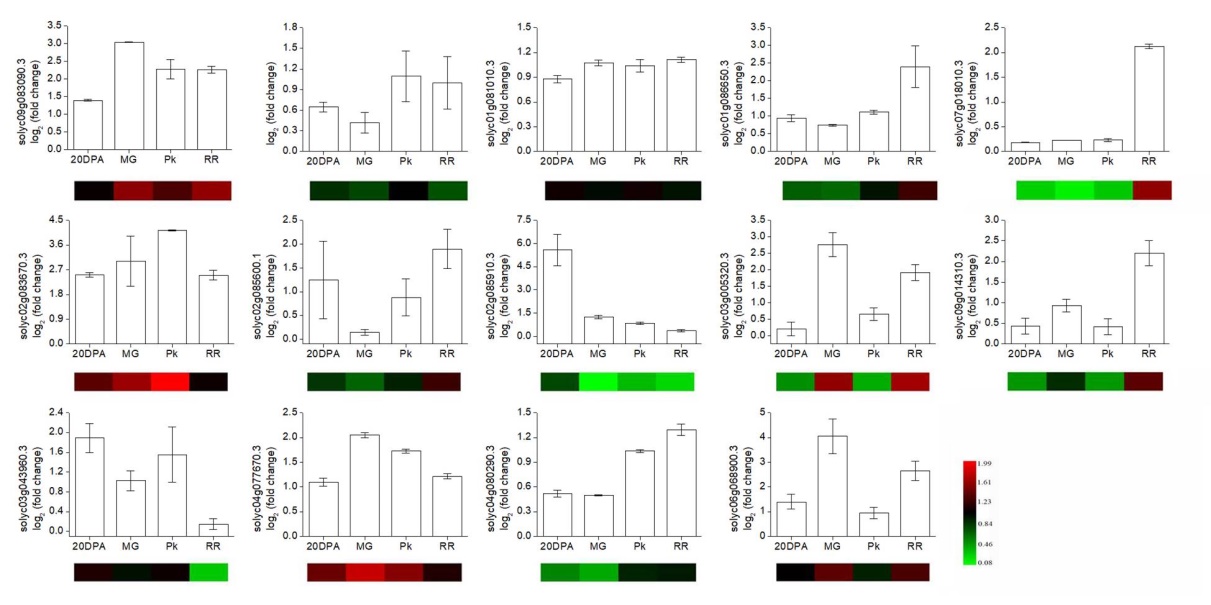


**Supplemental Figure S6.** qRT-PCR analysis of some selected genes. The vertical axis indicates the log_2_(fold change) values. Fold change represents the fold change expression level of genes in OE-3 fruits compared with WT fruits.


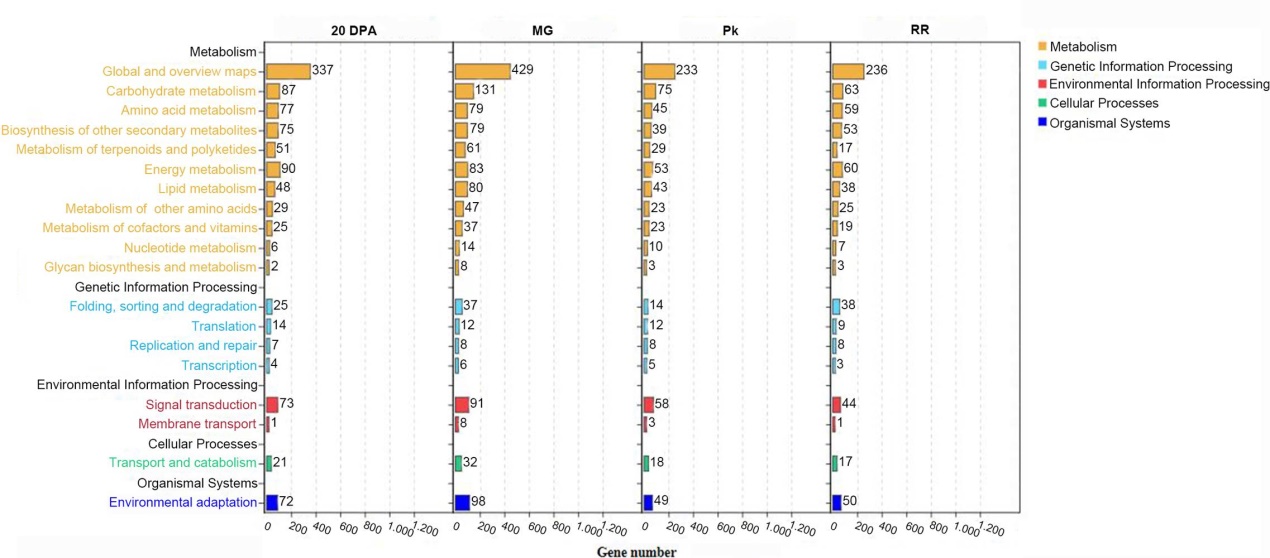


**Supplemental Figure S7.** KEGG pathway analysis of DEGs. The horizontal axis indicates gene number, while the vertical axis indicates pathway name.


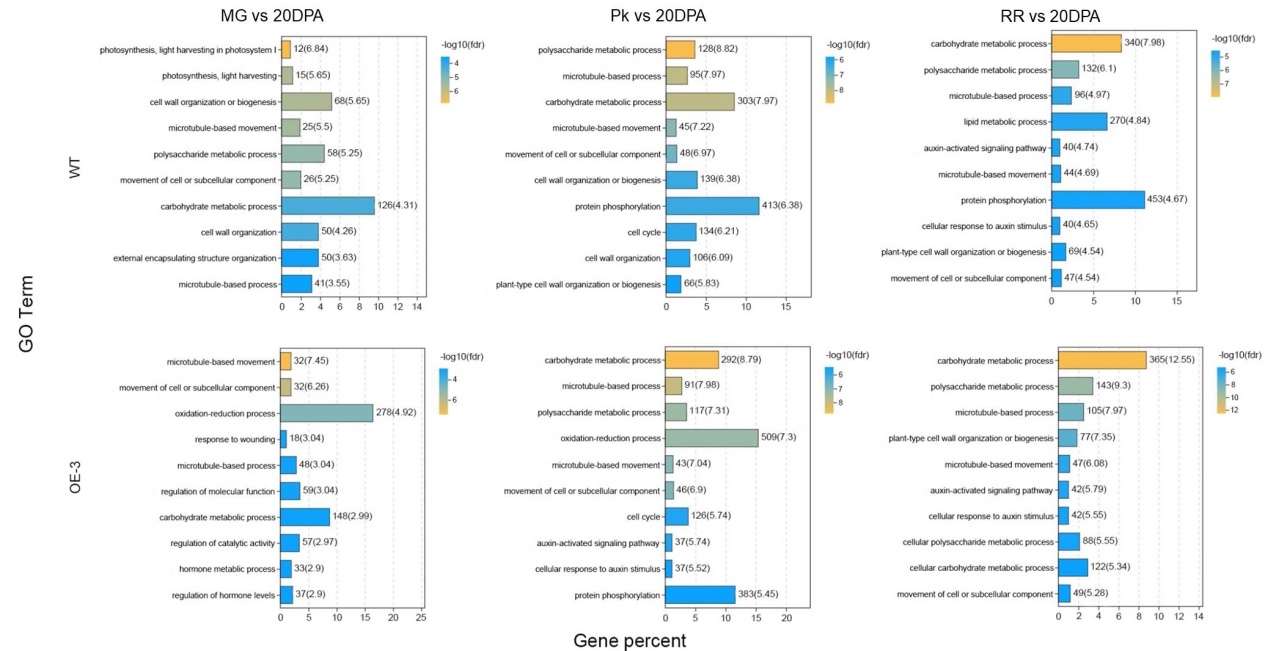


**Supplemental Figure S8.** GO analysis of DEGs. The horizontal axis indicates gene percent, while the vertical axis indicates GO Term. The top 10 significantly enriched GO terms were selected.
